# Application of natural orifice transluminal endoscopic surgery with ENDOCRAB system for stomach perforation model: ex vivo porcine study

**DOI:** 10.1038/s41598-024-56484-6

**Published:** 2024-03-27

**Authors:** Han Jo Jeon, Hyunjoon Hwang, Dokwan Lee, Yoonjin Kim, Jae Min Lee, Eun Sun Kim, Bora Keum, Yoon Tae Jeen, Hong Sik Lee, Hoon Jai Chun, Hyuk Soon Choi, Yongnam Song

**Affiliations:** 1 Division of Gastroenterology and Hepatology, Department of Internal Medicine, Korea University College of Medicine, 73, Goryeodae-ro, Seongbuk-gu, Seoul, 02841 Republic of Korea; 2 Department of Mechanical Engineering, Korea University, Seoul, Republic of Korea

**Keywords:** Gastrointestinal models, Stomach, Oesophagogastroscopy, Therapeutic endoscopy, Preclinical research, Mechanical engineering

## Abstract

Iatrogenic stomach perforation is a detrimental, irreversible, and fatal condition. Traditional surgery and endoscopic suturing clips and devices have been introduced to seal holes and prevent sepsis and disease progression. However, the development of endoscopic devices for perforations remains challenging, with no standard device available. This study investigates the superficial layer approximation strengths of the newly designed ENDOCRAB system for gastric wall defects. Thirty porcine stomachs were prepared ex vivo for the perforation model and distributed equally into three groups: ENDOCRAB system, Through-the-Scope Clip (TTSC), and hand suturing (HS). Both ENDOCRAB and TTSC achieved mucosal–submucosal layer apposition, whereas HS allowed a full-thickness layer. Their air leakage pressure and procedural duration were measured. The analysis of air-leakage pressure demonstrated comparable suture strength between ENDOCRAB (118.5 ± 41.7 mmHg) and HS (127.4 ± 30.2 mmHg, *P* = 0.812), but inferior strength with TTSC (73.6 ± 21.6 mmHg, *P* = 0.012). HS achieved the shortest procedural duration, whereas ENDOCRAB and TTSC showed no significant differences. The ENDOCRAB system showed significantly greater strength than the TTSC, was comparable to HS in strength, and required a procedural duration similar to that of the TTSC. Furthermore, long-term in vivo experiments and histological evaluations are essential.

## Introduction

Perforation within the gastrointestinal tract (GIT) involves structural disruption that extends through all layers. The estimated annual rate of perforations has increased owing to the popularity and expansion of endoscopy^1^. GIT perforation is a rare and unexpected life-threatening complication associated with a high mortality rate (6–33%)^2^. Furthermore, perforations in the stomach predominantly arise from the worsening of preexisting conditions (peptic ulcers, obstructions, and malignancies), anastomotic leakage after gastrectomy, and iatrogenic causes, including endoscopy-related procedures [endoscopic mucosal resection (EMR), endoscopic submucosal dissection (ESD), polypectomy], and foreign bodies. Secondary peritonitis caused by perforation is accompanied by intense pain, potentially leading to septic shock, and presents with difficulties in achieving spontaneous resolution. Following GIT perforation, patients often experience deteriorating consequences that frequently require primary surgical intervention.

With the development of minimally invasive endoscopic techniques, the widespread adoption of curative R0 resection for early GIT cancer has replaced invasive surgical approaches^3^. However, intraoperative or delayed perforations during procedures require endoscopists to have immediate management devices and skills. Under these technical circumstances, through-the-scope clips (TTSCs) are currently the primary tools used to close perforations via endoscopy. TTSCs have demonstrated a clinical success rate of 98.3% for stomach perforations and a 100% success rate for colon perforations^4^. While TTSC is effective for small perforations (< 1 cm), its ability to address larger perforations and fistulas is limited by factors such as the superficial grasp layer and the need to achieve stable apposition, proper approximation of defect margins, appropriate jaw compression forces, grasp depth of the superficial layer, and suitable opening width of the clip jaws^5^. Even after attempting TTSC, perforations that fail require conventional surgeries.

The recently introduced Natural Orifice Transluminal Endoscopic Surgery (NOTES) is an emerging minimally invasive technique that transcends the borders of traditional surgery and bridges the gap between surgical and endoscopic repair. Over-the-scope Clips (OTSCs; Ovesco Endoscopy, Tübingen, Germany) used in NOTES are novel endoscopic metallic clips. OTSCs enable the capture of large volumes of tissue and facilitate perforation management. They demonstrated non-inferior leak pressures compared with suturing in an ex vivo animal study^6^. The comprehensive clinical success rate of OTSCs was 78%, with a success rate of 85% for perforations, and a corresponding complication rate of 1.7%^7^. Caution is necessary for potential complications associated with OTSC use, such as luminal stenosis, micro-perforation, and abnormal suctioning of neighboring organs associated with OTSC usage^8^. Additionally, the constraints of the OTSC include the difficulties associated with its substantial cap size, navigating acute angles within the GI tract lumen, and restricted maneuverability.

Currently, perforation-closure devices are divided into two primary categories: sutures and clips. The OverStitch system (OverStitch and OverStitch Sx; Apollo Endosurgery, Inc., Austin, TX, USA) is a prominent endoscopic suturing system that enables full-thickness closure of large perforations (> 1 cm). In a recent multicenter prospective study, OverStitch demonstrated promising outcomes, with an overall technical success rate of 99.3% and a clinical success rate of 89%^9^. The unresolved issues include technical challenges and product availability in markets beyond the USA. Several other suture devices have been designed; however, most are suitable only for closing small defects (< 2 cm)^10^.

Wound closure devices should be user-friendly, effective, and capable of closing large wounds^11^. The perforation managing criteria may vary according to the guidelines, TTSC for gastric perforation size less than 1–2 cm is a currently recommended method with convenient manipulation, excellent perforation healing, effective leak sealing, and peritonitis prevention^12,13^. Even with its focus on opposing the superficial layer, the TTSC has demonstrated effectiveness in closing perforations. By combining the strengths of TTSC and their continuous suturing capabilities, a newly designed endoscopic suturing device can enhance both ease of use and effectiveness. Continuous suturing is superior to interrupted suturing for skin closure in terms of tensile strength and reduced dehiscence, and similar benefits have been anticipated for stomach tissue based on previous studies^14^. Furthermore, full-thickness suturing provides greater suture strength compared to superficial layer suturing^15^.

Although suturing devices have been used for full-thickness perforation closure, research on the effectiveness of continuous rather than interrupted suturing, specifically in the superficial mucosal–submucosal layer, is limited. Therefore, this study hypothesizes that when comparing the suturing tightness of defect closure completed with the same number of attempts, the mucosal-submucosal apposition method, ENDOCRAB with continuous suturing, is expected to be stronger than the conventional TTSC, which employs an interrupted technique, but is weaker than hand suturing, which involves full-thickness interrupted suturing. This study compared the feasibility and effectiveness of mucosal–submucosal continuous suturing using a newly designed device with a TTSC and manual suturing in an ex vivo stomach wall perforation model.

## Materials and methods

### Animal preparation

Porcine stomachs were harvested at the slaughterhouse within 24 h of death and used to evaluate tensile strength using suturing methods. The pigs used in the study had an average weight of 50 kg and were 6 months old. The pigs were raised on farms in Korea (Gyeonggi-do, Korea) for food. Porcine stomachs were immediately frozen and thawed prior to experimentation. The interior of the stomach was rinsed with tap water before each suturing method was applied.

### Ethical approval

None of the pigs was euthanized for experimental purposes. Given that this study used stomachs obtained from pigs reared for meat, the requirement for ethical approval was waived by the Institutional Animal Care and Use Committee of Korea University College of Medicine.

### Ex vivo stomach perforation model

**Figure 1:**
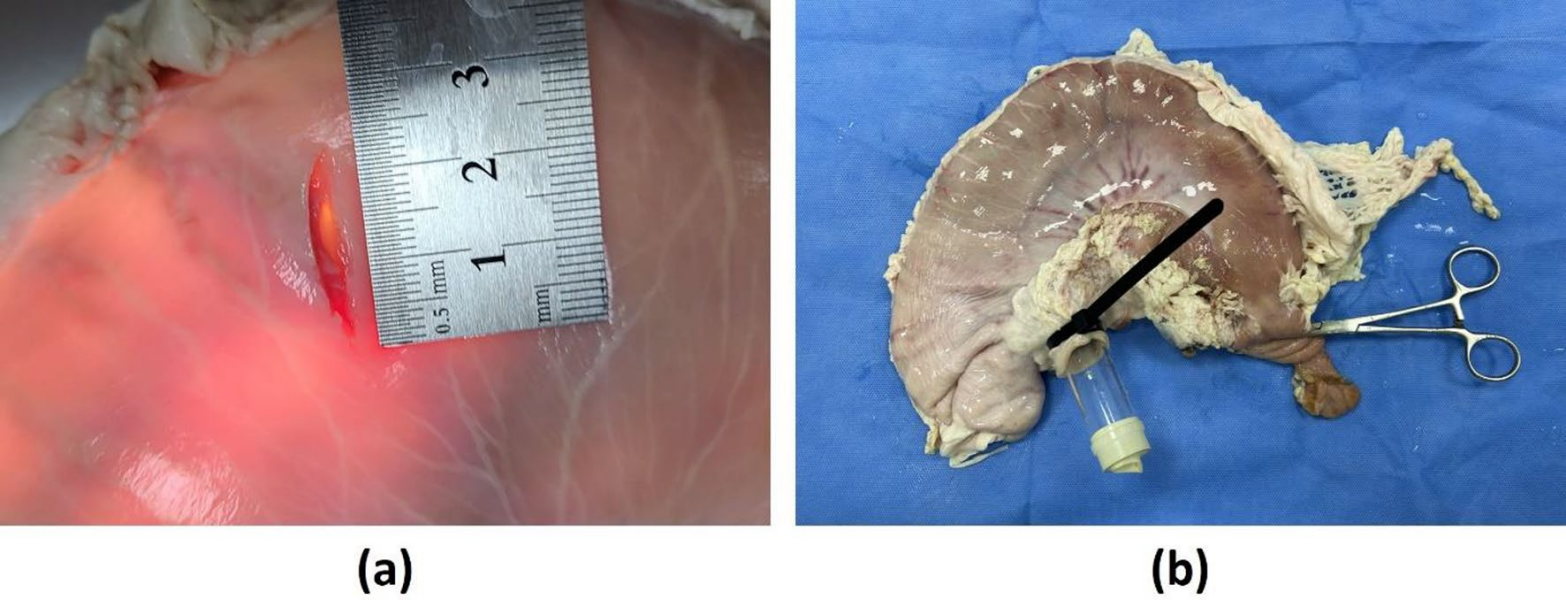
Stomach perforation model: (**a**) a 20-mm-sized incision was artificially made on the anterior wall of the lower gastric body; and (**b**) the two passages of the stomach (the duodenum and the esophagus) were sealed with a Kelly clamp and a latex-wrapped acrylic tube, respectively, to prevent the leakage of air.

The first portion of the duodenum and the distal esophagus (approximately 3 cm) connected to the stomach were harvested simultaneously. Stomachs showing serosal damage were excluded from this study. A vertical 20-mm-sized incision was made artificially on the anterior wall of the lower gastric body, where there exists a high risk of perforation during the procedure, as well as easy and reliable endoscopic access, using a surgical blade (Fig. 1). All incisions were made at the same location, and each stomach had a single incision. A Kelly clamp was used to clamp the duodenum and prevent air leakage during endoscopic procedures. An 18-mm-diameter cylindrical acrylic tube was inserted into the esophagus and fixed with cable ties. The latex was wrapped and affixed to the oral side of the tube where the endoscope was inserted, and a 0.5 cm-sized hole was introduced to facilitate free movement of the endoscope. Subsequently, an endoscope (GIF-Q260; Olympus, Tokyo, Japan) was inserted through the acrylic tube, and the air was insufflated to establish a stable working space.

### Study group allocation

**Figure 2:**
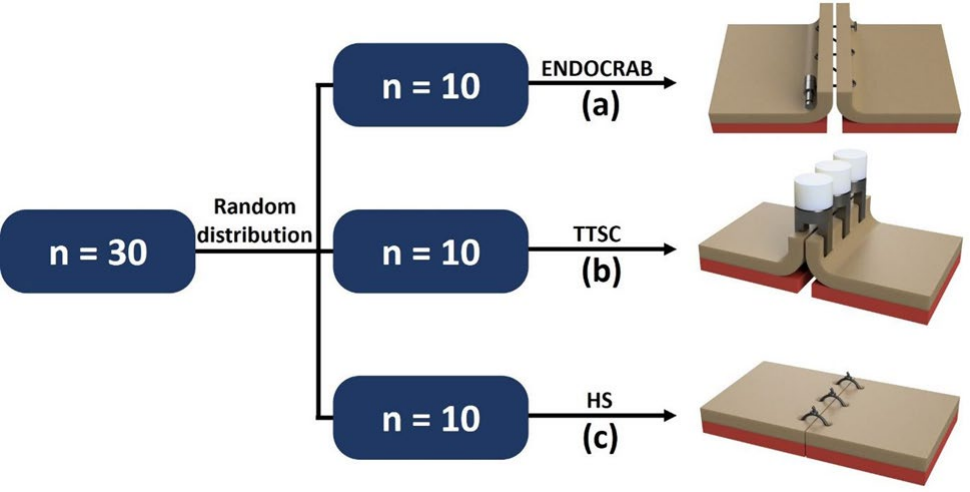
Sample distribution and schematics of each closure technique: A total of 30 porcine stomach samples were prepared, with 10 samples randomly assigned to each of the following groups: (**a**) ENDOCRAB, (**b**) Through-The-Scope clips (TTSC), and (**c**) Full-thickness hand sutures. In the case of the hand suture, full-thickness closure was performed, while the ENDOCRAB and TTSC were used for mucosal–submucosal layer closure.

The collected stomachs were randomly distributed into three suturing groups: the ENDOCRAB system, TTSC, and hand suturing (Fig. 2). These three groups comprised two experimental groups (ENDOCRAB system and TTSC) and one control group (hand suturing). Depending on the suturing thickness, the depth of mucosal–submucosal suturing was used in the ENDOCRAB system and TTSC, whereas the hand-suturing technique utilized a full-thickness gastric wall, which is the most robust method of suturing. Furthermore, both the ENDOCRAB system and the TTSC were used to compare the differences in suturing strength between the device and the standard clip.

### Full-thickness hand-suturing

The hand suture method was used to simulate a simple closure for stomach perforation. A simple interrupted stitch was used to close the defect in accordance with a previous study^16^. In this group, the perforated stomach was sutured with a full-thickness layer of 4–0 surgical nylon thread (52-R-NB4040; Deknatel Inc., New York, NY, USA), an identical thread used in the ENDOCRAB system. Three interrupted sutures closed the 2.0-cm dehiscence line^17^. The hand-suturing method was initiated at the central point of the perforated line in the serosal layer. Sequential apposition of the upper and lower margins of the defects was completed, with the central suture serving as an anatomical reference point. Spacing (1.0 cm) was maintained between the sutures. Three consecutive knots were used. After the first double knot, two additional single loops are incorporated similarly to hold the knot. Each loop was drawn in the opposite direction along the wound edge.

### Endoscopic clip

Endoscopic clips (EZ clips; HX-610-135L, Olympus) were used to close the holes. After the endoscope was inserted into the stomach, its tip was placed at the perforation site. The central point of the incision line was initially approximated using TTSC, followed by the sequential application of clips at the edge of the perforation, maintaining a 1.0 cm spacing between the initial clipping sites. The stomach perforation was completely secured using three endoscope clips. Displaced TTSCs were immediately removed according to the endoscopist’s decision, followed by repeated clip placement to ensure tight closure. Endoscopic clips with a poor approximation of the mucosal layer were discarded, and only those that achieved successful symmetrical mucosal approximation on both sides based on perforation were used for pressure measurement.

### Endoscopic successive suturing device (ENDOCRAB system)

**Figure 3:**
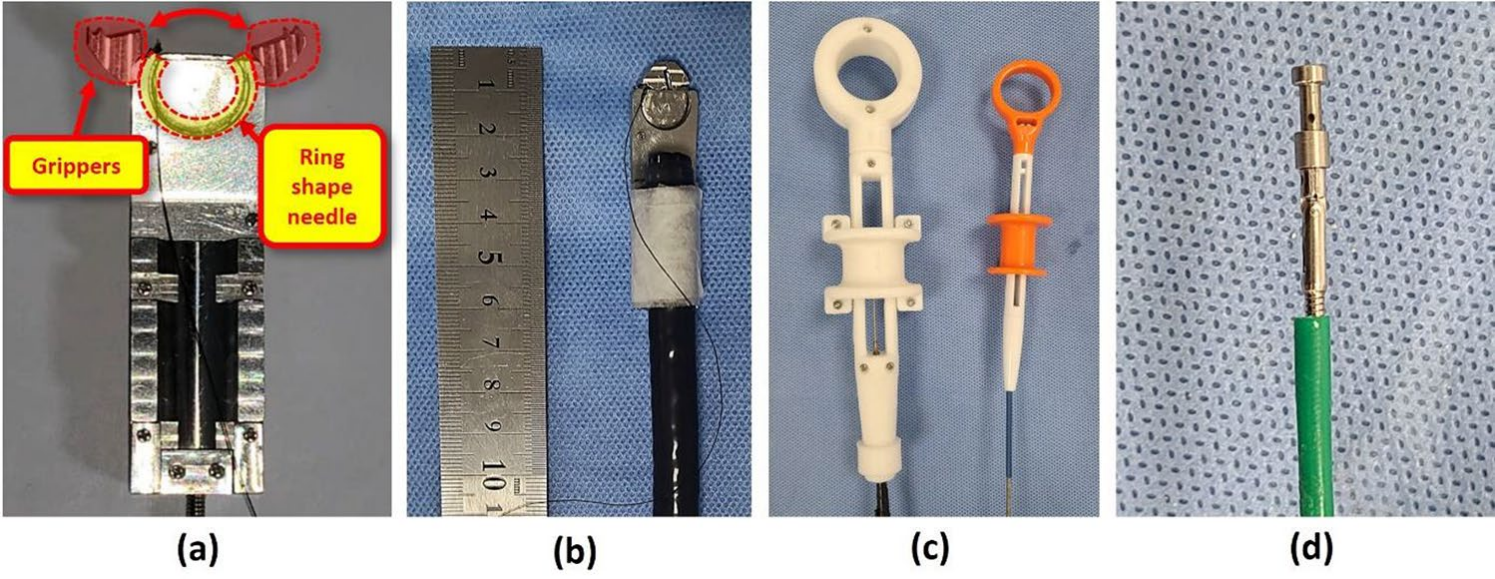
Composition and appearance of the ENDOCRAB system: (**a**) The system comprises grippers that secure the tissues to be sutured and a ring-shaped needle mounted on the main body, which penetrates the tissues. (**b**) The appearance of the ENDOCRAB attached to the endoscope’s tip. (**c**) Two controllers of the ENDOCRAB system (left: rotates the needle, right: opens and closes the grippers). (**d**) The knotting device is held with modified forceps.

The ENDOCRAB system consists of grippers, a main body, two handles, additional equipment, and a knotting device (Fig. 3). The metallic body of the ENDOCRAB system was made of stainless steel and was 50 mm in length, 15 mm in width, and 5.45 mm in thickness. The gripper, located at the forefront of the ENDOCRAB system, is composed of two jaws (each 6 mm in size) connected to an endoscopic handle through wires, which enables selective tissue capture. Upon opening the gripper, the ring-shaped needle (diameter: 13 mm) within the body became visible and was manipulated using an additional handle through another wire. Song et al. reported that a knotting device comprising a male stud and female ring was controlled by modified conventional endoscopic forceps surrounded by a flexible outer tube^18^. The main body was affixed to the endoscope tip using skin tape. A ring-shaped needle was threaded with a 4–0 surgical nylon thread (52-R-NB4040; Deknatel Inc., New York, NY, USA), and the bead was fixed at the end of the thread. Both ENDOCRAB and knotting devices are protected by patents (DP20220544, DP20220545, DP20220603, and DP20120310, Korea).

**Figure 4:**
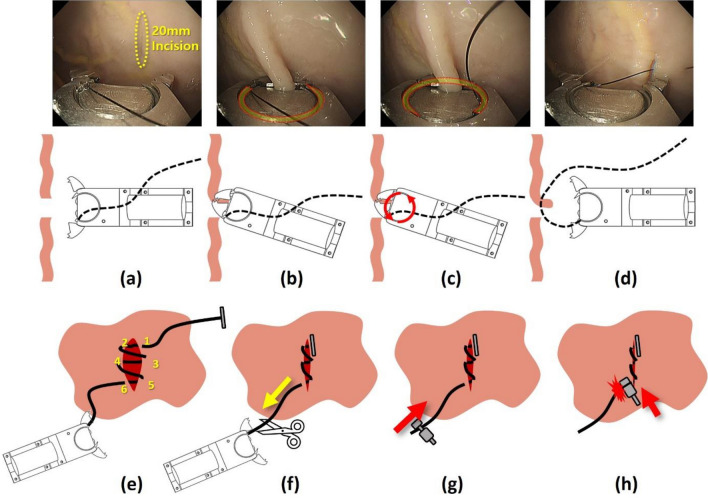
Closing procedure of the ENDOCRAB system: (**a**) Open the grippers to start suturing. (**b**) Carefully close the grippers to hold the tissue to be sutured. (**c**) Rotate the ring-shaped needle 360° through the grippers to penetrate the fixed tissue. (**d**) Open the grippers and repeat the previous steps to complete the closure loop. (**e**) Withdraw the endoscope carefully after completing six penetrations. (**f**) Cut the connected suture thread. (**g**) Pass the thread through the knotting device and gently push inward to locate the knotting device in the appropriate position. (**h**) Pushing the female stud of the knotting device securely fixes the knotting device, and the remaining thread is cut. Then pull out the cut thread.

The study protocol involved three stitches and six penetrations to close the defect. The suturing process of the ENDOCRAB system is as follows (Fig. 4): (1) The incision site was targeted with the grippers and kept wide open; (2) one side of the gastric tissue was captured based on the incision by grasping the grippers. (3) The ring-shaped needle was rotated to pierce the stomach tissue; (4) the grippers were opened to release the tissue, and the next suture site was explored; (5) steps (1)–(4) were repeated six times to completely close the incision site; (6) the endoscope was withdrawn and the thread was cut between tissue and the ENDOCRAB system. (7) the thread attached to the tissue was passed through an oblique hole in the male stud of the knotting device; (8) the male stud was inserted into the female ring and held with modified forceps surrounded by an outer tube; (9) the knotting device was gently pushed inward with the endoscope to maintain a clear view of the surgical site; and (10) the knotting device was positioned along the thread at the suture site and firmly anchored by pushing the female ring to the end of the male stud. The remaining thread was then removed from the stomach (Supplementary Video).

### Measurement of air-leakage pressure

After closure of the perforation, the acrylic tube was removed and a PVC tube with a diameter of 10 mm was inserted through the esophagus and tightly sealed using a cable tie. The tube was connected to an air pump, while the Kelly clamp maintained grasping of the duodenum. A digital pressure meter (PS-9303SD; Lutron, Coopersburg, PA, USA) was connected directly to the PVC tube to measure the air pressure inside the stomach, which denotes the suture strength. The specimens were submerged in a water reservoir in a plastic box and compressed air was gradually injected into the stomach. Air pressure in each stomach was recorded when the first air bubble was detected.

### Study outcomes and definitions

The primary objective of this study was to evaluate the feasibility of the ENDOCRAB system in a stomach perforation model. The interruption of each suture method was assessed for feasibility. Procedure interruption was defined as any event that led to the cessation of the ongoing procedure. In cases of interruption due to misplacement of TTSC, the misplaced clip was removed, and a new clip was attempted for defect closure. When a malfunction or breakage occurred with ENDOCRAB, the suturing was temporarily interrupted before resumption. The duration of interruption was included in the total procedure time. Despite efforts to address interruptions, the inability to achieve defect closure was classified as a “failure”. Secondary outcomes included measurement of air leakage pressure for tissue tightness at the perforation site and procedure time. Based on the patterns of air leakage at the suture site, we identified two major types of leakage: spontaneous and explosive. The spontaneous type was defined as air bubbles escaping from the weakest of the three sutured sites. However, the explosive type featured burst tearing of the neighboring muscularis propria rather than the sutured site. The procedural duration was measured from the time the endoscope was inserted into the stomach to the time of withdrawal. Procedural duration in the hand-suture group was defined as the duration between piercing the serosal side and completing the third suture.

### Statistical analysis

All statistical analyses were performed using the IBM SPSS Statistics version 27 (IBM, Armonk, New York, USA). Shapiro–Wilk and Levene’s tests were conducted to confirm the normal distribution and homogeneity of variance. One-way ANOVA was conducted to examine the differences among the groups and to investigate the differences between each pair of groups. Tukey’s honest significant difference test was employed in cases where the homogeneity of variances was satisfied, whereas the Dunnett T3 test was used in cases where the homogeneity of variances was not satisfied. Continuous variables were expressed as means and standard deviations, while categorical variables were presented as percentages. The comparison of air leakage pressure between explosive and non-explosive types was conducted using the nonparametric Mann–Whitney U test and expressed as the median and interquartile range. Statistical significance was set at *P* < 0.05.

## Results

Thirty porcine stomachs were collected and randomly assigned to three groups: endoscopic clip, hand suture, and ENDOCRAB system, each consisting of 10 stomachs. All 30 procedures were completed defect closure in each group. The total interruption rate was 20% (6/30, TTSC: 4/10, 40%; ENDOCRAB: 2/10, 20%). All four cases of TTSC were due to inaccurate clip placement, while with ENDOCRAB, one case involved thread breaking and the other was due to mechanical malfunction. The control group underwent full-thickness repair with hand sutures, whereas the experimental group underwent mucosal and submucosal layer suturing using two endoscopic procedures: TTSC (interrupted suture) and ENDOCRAB (continuous suture).

**Figure 5:**
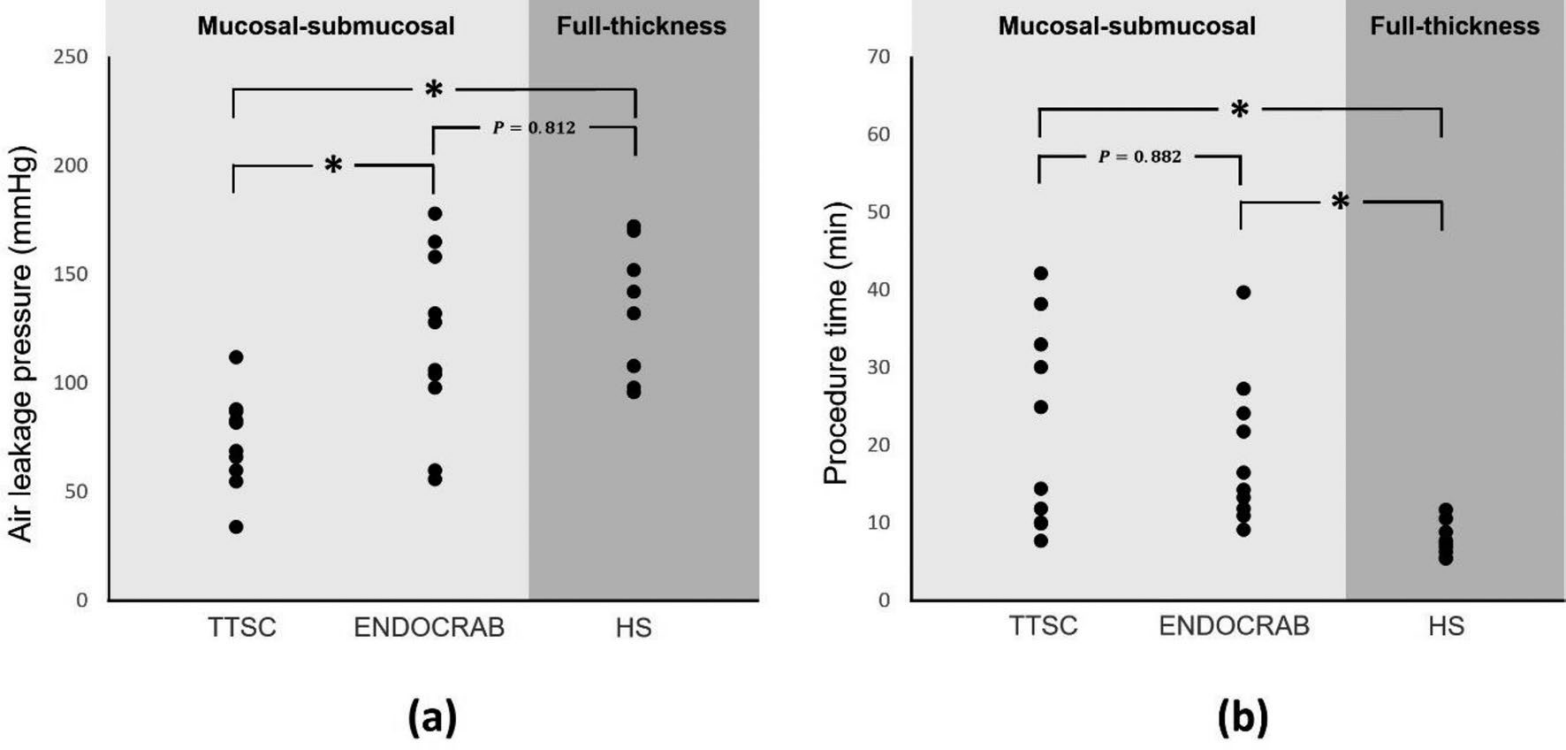
Comparison of each closure technique: (**a**) The mean ± standard deviation of the air leakage pressure measured was 73.6 ± 21.6 mmHg, 118.5 ± 41.7 mmHg, and 127.4 ± 30.2 mmHg for Through-The-Scope clips (TTSC), ENDOCRAB system, and full-thickness hand suture (HS), respectively. (**b**) The mean ± standard deviation of the procedure time was 22.2 ± 12.9 min, 18 ± 9.5 min, and 7.80 ± 2.1 min for TTSC, ENDOCRAB system, and HS, respectively. *P* value less than 0.05 was considered significant.

The air-leakage pressure in each stomach for different closure techniques is shown in Fig. 5a (Supplementary Table S1). The average air-leakage pressure was 73.6 ± 21.6 mmHg for TTSCs, 118.5 ± 41.7 mmHg for ENDOCRAB system, and 127.4 ± 30.2 mmHg for hand sutures (*P* = 0.002). The results showed that the air leakage pressure of stomachs closed using the ENDOCRAB system was significantly higher than that of stomachs closed using the TTSCs (*P* = 0.012). However, there was no significant difference in air leakage pressure between the ENDOCRAB system and hand sutures (*P* = 0.812).

The average procedural duration was 22.2 ± 12.9 min for TTSCs, 18 ± 9.5 min for ENDOCRAB system, and 7.80 ± 2.1 min for hand sutures (*P* = 0.005) (Fig. 5b), (Supplementary Table S2). The results indicated that the procedural duration for hand suture closure was significantly shorter than that for both the TTSCs and the ENDOCRAB system (clip–ENDOCRAB system, 0.882; clip–hand suture, 0.018; ENDOCRAB system, hand suture, 0.014). However, there was no significant difference in the procedural duration between the ENDOCRAB system and TTSCs (*P* = 0.882).

**Figure 6:**
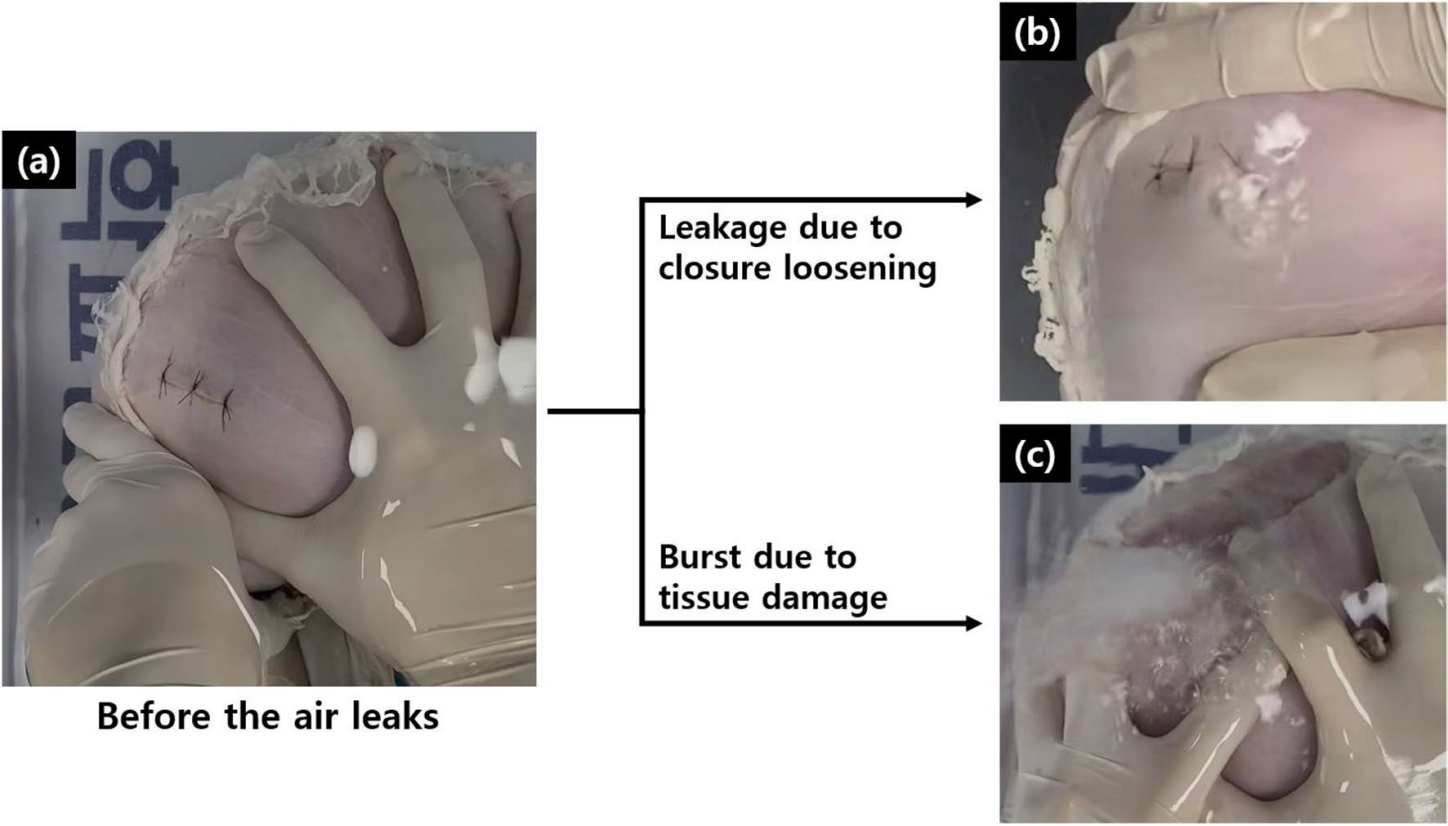
Types of air leakage: (**a**) An inflating closed perforation model before the air leaks. (**b**) In the non-explosive type, where the sutured ties of the incision loosen, air escapes through a slightly separated gap. (**c**) In the explosive type, air escapes as if bursting when the tissues around the suture sites tear.

**Figure 7:**
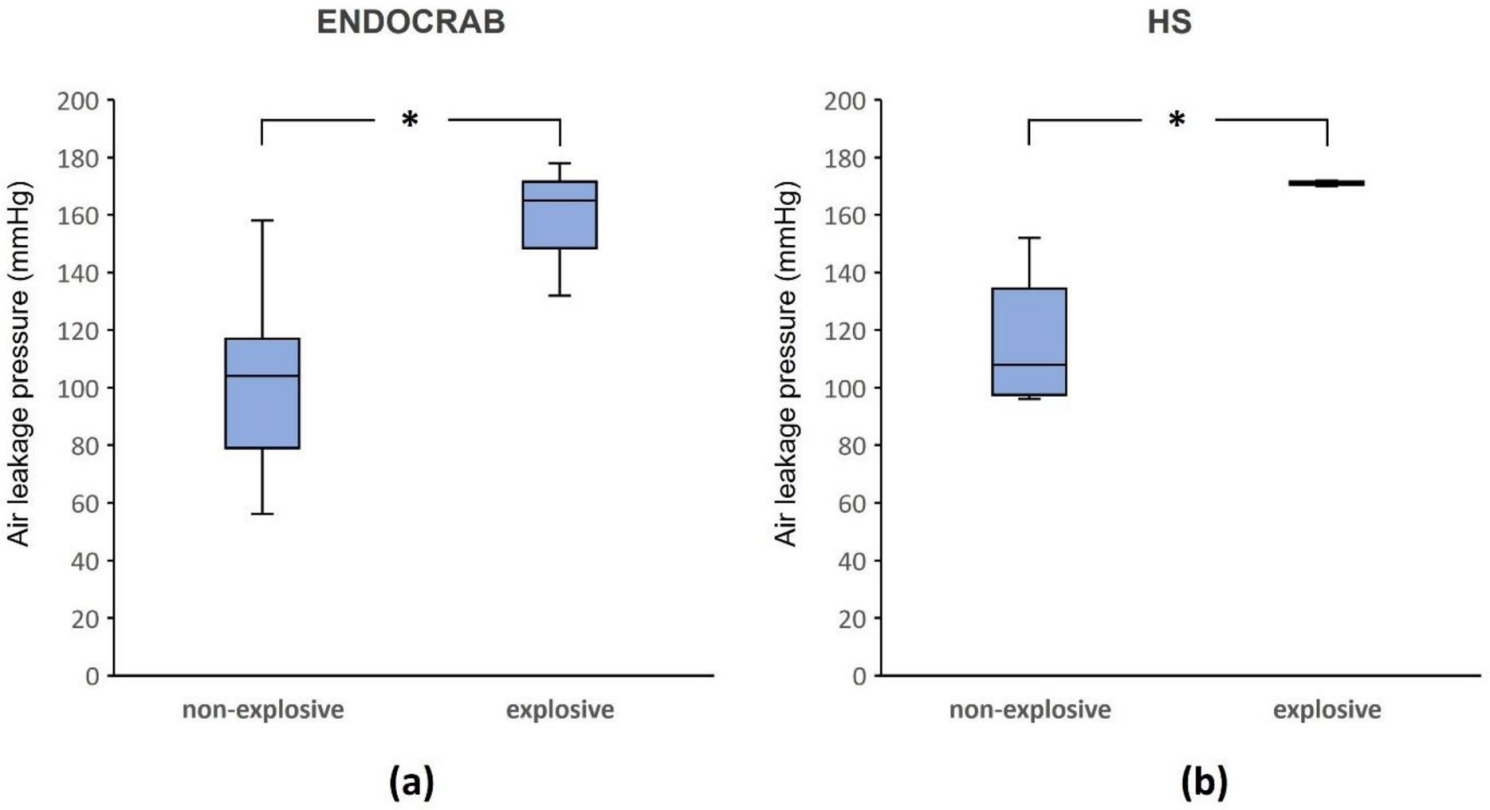
Subgroup analysis based on air leakage type: (**a**) In the case of the ENDOCRAB, a significant difference was found between the two air leakage types (*P* = 0.03). (**b**) Similarly, within the hand suture (HS) group, a significant difference was observed between the two air leakage types (*P* = 0.036). *P* value less than 0.05 was considered significant.

As the pressure increased, most of the sutured ties at the incision sites weakened and showed slight separation, resulting in the formation of air bubbles (Fig. 6). However, some stomach tissues exhibited burst tearing of surrounding tissues near the suture sites. No tissue tearing was observed in the TTSC group. The ENDOCRAB and hand suture groups demonstrated tearing rates of 30% and 20%, respectively (ENDOCRAB [30%]: 158.3 ± 23.7 mmHg; hand sutures [20%]: 171.0 ± 1.4 mmHg). A significant difference was observed between the explosive and non-explosive types within the ENDOCRAB system (*P* = 0.03) (Fig. 7). Additionally, within the hand suture group, the explosive type showed a significant difference compared to the non-explosive type (*P* = 0.036). However, when comparing the explosive types, there was no significant difference between the ENDOCRAB system and hand suture groups (*P* = 1.0).

## Discussion

This is the first ex vivo study using ENDOCRAB system to compare and elucidate the strength of superficial mucosal–submucosal layer continuous apposition, rather than interrupted TTSC and full-thickness suturing in the porcine stomach perforation model through air-leakage pressure testing. In this trial, we introduced a newly designed ENDOCRAB system for the superficial layers to ensure safety and convenience. Our primary outcome was to determine the feasibility and applicability of the stomach perforation model. The secondary outcome included ascertaining whether superficial layer apposition could achieve tissue tightness comparable to that achieved with full-thickness sutures. To compare the closure strength of ENDOCRAB with that of the conventional TTSC and hand suture methods, we examined the air leakage pressure of the sutured perforations in each method. Although the ENDOCRAB system only opposed the mucosal and submucosal layers, the tissue tightness surprisingly compared closely with that of the hand suture method using full-thickness sutures. The TTSCs showed statistically lower strength and sustainability than the ENDOCRAB system. This can be attributed to the unique ENDOCRAB system, which encompasses further tightening of the loose thread during continuous suturing and bead placement after completion of suturing. These findings suggest that the ENDOCRAB system has the potential to establish a new superficial suture endoscopic instrument in addition to the full-thickness endoscopic suture device, as there is no standard device for suturing the mucosal layer, although several methods have been introduced^19^. Moreover, the ENDOCRAB system can be considered a favorable alternative to TTSCs, offering improved effectiveness and enhanced tissue approximation forces, making it a promising option for managing mucosal defects.

In accordance with our study, Matsui et al. previously reported the results of a comparison of mean air-leak pressure between the purse-string method utilizing endoloop with clips for mucosal closure (13.7 ± 3.35 mmHg) and OTSC for seromuscular closure (24.8 ± 3.13 mmHg)^20^. The outcome of the air-leak test demonstrated a high strength of seromuscular closure compared to mucosal closure (*P* < 0.01), which correlated with the superior strength of hand suturing in the present study compared to the TTSC. The outcomes of both abovementioned studies can be predicted to some extent. This is because the entire layer is thicker and has more robust tissue tensile strength than the mucosal layer. However, an interesting observation of the traction tension test could be emphasized on OTSC (mucosal layer, 18.3 ± 2.16 N) exhibiting a statistically lower strength than surgical hand suture (whole layer, 62.4 ± 7.26 N), which was contrary to our main results exhibiting the leakage pressure of ENDOCRAB (mucosal–submucosal layer, 118.5 ± 41.7 mmHg) was comparable to that of surgical hand suture (whole layer, 127.4 ± 30.2 mmHg). Although the ENDOCRAB system in our study repaired only the mucosa–submucosa layer, the continuous suture method and anchoring of the bead to the tissue for firm tightening yielded a closure strength similar to that achieved by the full-thickness suture group.

Our primary finding aligns with that of prior endoscopic hand-suturing (EHS) research^21^, which utilized an EHS system allowing continuous suturing to close the defect, and compared it with conventional endoscopic clips and those using the detachable snare method. The results showed that the EHS demonstrated the highest suturing strength among the three mucosal suturing techniques. EHS research has focused exclusively on comparing mucosal suturing methods; the full-thickness hand suturing method was not included in this comparison. What we have ascertained from this investigation and the EHS research is that, among the existing mucosal suturing methods, successive suturing surpasses other techniques in terms of strength, possibly even rivaling the hand suture method.

The key importance of our investigation lies in challenging the findings suggesting that superficial layer apposition exerts a lower force than muscular layer apposition. Another distinguishing factor between the two studies was the hand-suture method. One of the most standard suturing methods, interrupted simple sutures, was employed in our study, whereas Matsui et al. used Albert–Lembert sutures at 7-mm intervals for end-to-end anastomosis of two separated tissues^20^. Although interrupted hand sutures are more common and slower than continuous sutures, the reported rates of wound dehiscence were similar between the two groups^22,23^. Moreover, many types of suture methods and materials are available, even for interrupted sutures. The closure strength of the hand suture may differ depending on the number and direction of square knots. Nonetheless, the fact that ENDOCRAB nearly matched the strength of the widely employed full-thickness sutures strongly suggests the clinical utility of superficial layer suturing.

In the case of endoscopic clips, air leakage occurred when the clip shifted slightly or was completely detached from its initial fixation point owing to pressure. However, for the ENDOCRAB system and hand sutures, the knotting device or bead remained intact, and the sutured loops continued to hold without detachment or breakage, enabling them to withstand relatively high pressures. Some specimens with exceptionally high air leakage pressures exhibited a phenomenon in which the surrounding tissue near the sutured area was damaged before the suture loop, leading to a burst explosion (ENDOCRAB, 30%; hand suture, 20%; TTSC, 0%).

Comparing the procedural durations, hand suturing required the shortest time (within 8 min), whereas clips and the ENDOCRAB system required approximately 22 and 19 min, respectively. The two mucosal suturing methods, TTSC and ENDOCRAB, demonstrated no difference in procedural duration. The TTSC struggles to achieve a balanced symmetrical mucosal approximation on both sides. After asymmetrical clipping, reattempts after mispositioned clip removal were necessary in 60% of cases. The ENDOCRAB system demanded a timeframe similar to that of the TTSC, which was attributed to the necessity of six repetitions in contrast to the three repetitions required for clipping. The measured procedure time in the hand suturing group could have been largely influenced by the ex vivo experimental conditions and suturing technique. Therefore, the interpretation that hand suturing requires a shorter procedural duration than other mucosal suturing methods requires caution.

Mucosal EHS was performed for 20–30 min to suture a defect of approximately 30 mm in both animals and humans^24^. There was no significant difference in mucosal suturing for a 20-mm defect between EHS and endolooping for large defects (19.7 vs 19.8 min)^21^. Endolooping, a suturing method for relatively large defects, may be inferior to ENDOCRAB because of the similar procedure times and powerful suturing forces of the ENDOCRAB system. Although both endoloops and ENDOCRAB offer the advantages of safety, speed, and minimal risk of adjacent organ damage, endoloops have the drawbacks of sudden deflation, ease to glide, the requirement of a two-channel endoscope, and potential pain, whereas ENDOCRAB requires attachment to an endoscope^25–28^. Consistent with a previous suturing method using endoclips (16 min) for a 16–18 mm incision ex vivo, our TTSC durations were similar (22 min)^29^. An important finding of this study is that the procedure time for OTSC was shorter than that for TTSC. Further investigation of the ENDOCRAB system compared with the OTSC is needed to clarify which method is more effective in terms of time.

The suturing device seves as an auxiliary system for endoscopists to address adverse clinical situations, including gastrointestinal tract wall defects, resulting from procedures such as EMR and ESD. For defects < 1 cm in size, TTSC is the recommended modality with a reported success rate of 99%. However, for perforations 1–3 cm in size, the current recommendation is to use OTSCs in combination with endoloops, with a reported success rate of 88.7%^12^. It is important to note that according to the European Society of Gastrointestinal Endoscopy guidelines, the use of new suturing devices is currently restricted to expert hospitals with strict limitations^12^.

Several endoscopic suturing devices for defect repair have been introduced and updated for their convenience and ease of use. The OverStitch system is a representative full-thickness suturing platform that ensures safety and a high clinical success rate. However, implementing reliable suturing at 3–4 mm intervals remains an obstacle because of the absence of supplementary tools to grip the defect wall after punctuation^30^. Another closure device for endoscopic full-thickness resection is Zeosuture M (Zeon Medical Co., Tokyo, Japan)^31^. Suturing was accomplished by affixing the endoscopic tip, positioning the structure to penetrate ahead of the tissue, and inserting the needle towards the front-facing tissue. The lack of a mechanism to hold and stabilize the suturing tissue area during penetration can increase the risk of damage to surrounding organs. The EnVision system (EnVision Endoscopy, Somerville, MA, USA) is an innovative endoscopic suturing device consisting of a distal attachment to a flexible endoscope, a circular needle, and proximal actuators^32^, which allows knots without independent grasping clamps. Furthermore, the mechanism of suturing large mucosal defects in EHS closely resembles that of the ENDOCRAB system used in this study, which utilizes a rotating circular needle. EHS requires an additional step involving clip fixation at the start and end of the thread as well as a two-channel endoscope with forceps and a needle holder^21,24^.

The aforementioned devices require separate tools, such as forceps, or equipment, such as a helix, to prevent the tissue from being damaged. In addition, most devices encounter insufficient real-time visualization of tissue penetration. However, the ENDOCRAB system incorporates a simple and user-friendly structure to secure the penetrated tissue at an appropriate thickness. ENDOCRAB was designed after the need arose to separate the equipment to stabilize the tissue while preventing potential damage to external organs. The curved distal end also allows direct observation during tissue penetration, thus enhancing procedural ease and reliability. The development of various procedures, such as ESD, peroral endoscopic myotomy, and EMR, has led to the intentional creation of large mucosal defects. Previous research on mucosal defect closure has shown that closed defects exhibited less neovascularity and fibroblasts in the submucosa compared to the unsutured group^33^. The newly devised ENDOCRAB system appears to overcome these challenges. Based on the mechanism of suturing both sides around the defect and closing it by tightening the suture thread, the precise and reliable suturing capabilities of the ENDOCRAB system may also facilitate secure mucosal defect closures. Further experiments for mucosal defect closure would be warranted for the employment of this new suturing system.

ENDOCRAB grippers play a crucial role in achieving precise closure by securely holding the tissue to be pierced and allowing control over the thickness of the tissue to prevent damage to surrounding organs. The device design features two straightforward operating systems, gripping and needle rotation, which are controlled by two wire controllers. These controllers require simple pull-and-push motions to enable easy and efficient suturing. Moreover, the tip of the ENDOCRAB was slightly bent upward, and the needle was positioned at the top layer, allowing for real-time observation of the needle and suture site during the procedure. These structures ensure the stability and reliability of the suturing process, as the endoscopist can continuously monitor and maintain control over the needle and suture sites throughout the procedure. Visualization and real-time feedback to endoscopists enhance the safety and trustworthiness of the device during the suturing process, providing a higher level of precision and confidence to the operator.

One remaining challenge with the ENDOCRAB system is the incomplete inverted closure, which raises questions regarding how long mucosal suturing can endure in the stomach. Clinical evidence suggests that tissue healing depends on the depth of the layer in which the sutures are placed^34^. In this regard, sutures do not last long, particularly when placed superficially. Goto et al. reported similar results in that suture closure of the EHS (defect size 30 mm, stitch no. 3–6) was not maintained for 1-week post-procedure. However, more stitched wounds (no. 6–7) and wider suturing (7.6 mm vs 4.8 mm) showed greater durability for 4 weeks after ESD^24^. This interpretation permits mucosal layer suturing because the study was performed without muscularis propria defects. However, our model was confined to muscularis propria defects. Hence, reviewing prior research comparing TTSC and OTSC conducted in an in vivo gastrostomy model, most TTSC closures involve the mucosa (80%), whereas OTSC closures extend to the submucosa (70%)^35^. While TTSC initially promotes mucosal–mucosal layer apposition (20 mm), histologically it exhibits a layer-to-layer healing process, ultimately achieving transmural healing (75%) with full muscular bridging (100%)^36^. Therefore, the superficial layer approximation of ENDOCRAB in this study was also considered to histologically induce complete healing and superior strength compared with TTSC, which is believed to reduce the risk of leakage or abdominal infection. Given these findings, future investigations involving the histological assessment of the ENDOCRAB system in an in vivo gastrostomy model are warranted.

Our study has several limitations. The location of the full-thickness defect in the stomach is limited to the body and anterior wall. The placement of the perforation was determined based on the previous study with its convenient accessibility for endoscopic approach and procedures in ex-vivo settings among the site where the iatrogenic perforation due to EMR, ESD most frequently occurred during endoscopy^37–39^. It is necessary to validate the suturing capability of ENDOCRAB by altering the placement of the defect, including the proximal and distal parts of the stomach. We compared the ENDOCRAB system, which in depth is limited to the submucosal layer, with hand suturing that involves all layers. In this trial, histopathological assessment was restricted because of the ex vivo experimental design. Further in vivo trials should guarantee a microscopic view of the repaired tissue sections using the ENDOCRAB system to identify the layers to which the defect adheres. Another limitation of the ENDOCRAB system is its inadequate gripper length, which prevents complete layer grasping and full-thickness suturing. Despite being restricted to holding the mucosal–submucosal layer, it attained tightness like that of hand sutures. Future research should envision that extending the length of the gripper can suture all layers of defects. Although the procedural duration for the TTSC and ENDOCRAB systems using endoscopy was closely modeled to imitate in vivo conditions, the hand-suture group encountered a different situation from that of suturing within a narrow intra-abdominal space, similar to real-world surgical conditions. Hence, the procedural duration could fluctuate depending on circumstances resembling actual surgical situations or the surgeon’s level of expertise. Because the study was conducted by a single operator, the wide acceptance of the results could be restricted. Thus, future research should involve two or more endoscopists to address reliability concerns.

## Conclusion

This ex vivo investigation of the closure strength of the stomach wall demonstrates the potential for superficial layer apposition using an endoscopic suturing device. Although the endoscopic suturing device showed an incomplete inverted closure, the tissue sutured using the ENDOCRAB system established a substantial air leakage pressure comparable to that achieved through full-thickness suturing. Given that the ENDOCRAB system presents an identical mucosal suturing method to the TTSC, ENDOCRAB proved to be superior in achieving tissue tightness. However, further in vivo histopathological studies are required to determine whether ENDOCRAB suturing can be performed without loosening.

### Supplementary Information

Supplementary Information 1.

Supplementary Video 1.

## Data Availability

All data generated or analyzed during this study are included in this published article and its supplementary information files.
