# Supplementary material for: Application of natural orifice transluminal endoscopic surgery with ENDOCRAB system for stomach perforation model: ex vivo porcine study
Source: Sci Rep. 2024 Mar 27;14:7289. doi: 10.1038/s41598-024-56484-6 (PMC10973374; doi:10.1038/s41598-024-56484-6)
Supplement: Supplementary file 1 — Supplementary Information 1. [file 41598_2024_56484_MOESM1_ESM.docx]

**Supplemental Data**

**Supplementary Table S1.** The complete set of air leakage pressure data for three groups; TTSC, ENDOCRAP and HS

TTSC: through-the-scope clip

HS: hand suture

| **No** | **Air Leakage Pressure (mmHg)** | | |
| --- | --- | --- | --- |
|  | **TTSC** | **ENDOCRAP** | **HS** |
| **1** | **34** | **56** | **108** |
| **2** | **112** | **98** | **132** |
| **3** | **60** | **178** | **96** |
| **4** | **88** | **60** | **108** |
| **5** | **82** | **158** | **96** |
| **6** | **55** | **104** | **172** |
| **7** | **83** | **106** | **152** |
| **8** | **69** | **128** | **142** |
| **9** | **87** | **165** | **170** |
| **10** | **66** | **132** | **98** |

**Supplementary Table S2.** The complete set of procedure time data for three groups; TTSC, ENDOCRAP and HS

TTSC: through-the-scope clip

HS: hand suture

| **No** | **Procedure Time (min)** | | |
| --- | --- | --- | --- |
|  | **TTSC** | **ENDOCRAP** | **HS** |
| **1** | **24.95** | **10.93** | **8.85** |
| **2** | **42.12** | **21.77** | **10.55** |
| **3** | **11.83** | **14.28** | **11.72** |
| **4** | **32.97** | **16.48** | **5.43** |
| **5** | **38.20** | **9.18** | **5.55** |
| **6** | **10.12** | **24.15** | **6.92** |
| **7** | **7.70** | **11.90** | **7.45** |
| **8** | **14.45** | **13.30** | **7.75** |
| **9** | **30.03** | **27.30** | **6.32** |
| **10** | **9.97** | **39.73** | **7.50** |

**Supplementary Video title**

Ex-vivo gastric perforation closure using ENDOCRAB

**Video legends**

Closing a 20mm gastric perforation in a porcine stomach using ENDOCRAB: Sewing and knotting procedure for mucosal-submucosal apposition
